# Dr. Carl Anton Ewald

**Published:** 1915-11

**Authors:** 


**Dr. Carl Anton Ewald** of Berlin, died September 20, aged 70. His book on Diseases of the Stomach, translated by Morris Manges for the first American edition in 1892, ran through several editions in German and English, and was the accepted model for nearly all other works on the subject. He was editor of the Berliner Klinische Wochenschrift and Librarian of the Berlin Medical Society. His position on the staff of the BUFFALO MEDICAL JOURNAL was admittedly honorary, in the sense that the acceptance of whatever tribute was thus paid to his professional achievements, honored the Journal. It would be an injustice to both parties, however, to imply that this position was merely nominal. Dr. Ewald had contributed to the original matter of the Journal a valuable paper, which was translated expressly for our readers, and he had, at various times assisted with advice and information. Several years ago, Dr. Ewald visited Buffalo and addressed the Academy of Medicine, surprising all by his fluent and correct English. We feel therefore, that in the death of Dr. Ewald, we must not only regret the passing of a distinguished medical scientist, author and clinician, not only acknowledge a personal association in special interests and their corresponding organization, but that we speak for a large number of the local profession, in lamenting the death of a genial gentleman whom we have known as a friend, though all too briefly.

